# Coitus induced vaginal evisceration in a premenopausal woman: a case report

**DOI:** 10.1186/1754-9493-5-6

**Published:** 2011-04-12

**Authors:** Nishikant N Gujar, Ravikumar K Choudhari, Geeta R Choudhari, Nasheen M Bagali, Mahendra B Bendre, Santosh B Adgale

**Affiliations:** 1 Department of General Surgery, Al Ameen Medical College, Bijapur, Karnataka, India; 2 Department of Gynecology and Obstetrics, Al Ameen Medical College, Bijapur, Karnataka, India; 3 Department of Pathology, Al Ameen Medical College, Bijapur, Karnataka, India; 4 S.R.T.R Medical College, Ambejogai, Maharashtra, India

## Abstract

Vaginal evisceration in premenopausal women after trans-abdominal hysterectomy is extremely rare in occurrence and only few cases have been documented in worldwide literature. Here we report a premenopausal woman with coitus induced trans-vaginal evisceration who had undergone trans-abdominal hysterectomy two years ago.

This article highlights coitus as a trigger event for inducing vaginal evisceration and that vaginal evisceration caused by sexual intercourse should be considered in the field of surgery when a pre-menopausal woman presents with acute abdominal pain with no history of any other traumatic episode.

## Background

Hysterectomy is an extremely common procedure performed routinely on a global basis with recognized complications which include infection, bleeding, bladder injury and prolapse of the vaginal vault [1].

Vaginal evisceration after transabdominal hysterectomy in a pre-menopausal patient with vault rupture and prolapse of small bowel during sexual intercourse is an extremely rare event, and when it occurs, it is a surgical emergency [2,3].

Since the first report in 1864 by Hyernaux, 113 cases have been reported in medical literature [4]. Joy et al. identified 12 cases of vaginal cuff evisceration resulting from coitus and 9 of these cases were post-vaginal hysterectomy [3].

In premenopausal cases, evisceration appears to be rarer and tend to be associated with either sexual or obstetric trauma, while in post menopausal women, it is usually associated with atrophic vaginal wall which have an increased risk of rupture [1].

Here we report a case of coitus-induced vaginal evisceration in a pre-menopausal female patient, with prolapse of around 1 meter of small bowel through the vagina; who had undergone abdominal hysterectomy two years back for dysfunctional uterine bleeding (DUB).

## Case Presentation

A 40 year old, South Indian, premenopausal female with an obstetric score of P_2 _L_2 _presented with a progressive lower abdominal pain from 4 days, a feeling of something giving way in the vagina during bowel motion from 4 days and a mass protruding through her vagina from 3 days.

The pain had become progressively worse, was constant in nature and accompanied by nausea.

Her last sexual intercourse had been 4 days earlier, at which time she had experienced lower abdominal discomfort and slight vaginal bleeding. She denied any unusual or aggressive sexual intercourse or use of sex toys.

Her reproductive history was unremarkable for 2 vaginal deliveries with no evidence of obstruction or prolongation.

Her past medical history consisted of an uncomplicated abdominal hysterectomy for dysfunctional uterine bleeding (DUB) 2 years back.

Other medical history included only mild asthma and her medication was asthalin inhaler.

**General examination **revealed a lady of normal weight. She was febrile and had a pulse rate of 128 beats per minute with blood pressure of 90/60 mm Hg. Her respiratory rate was 18 cycles per minute.

Her abdominal examination revealed signs of peritonitis. All hernial orifices were clear.

CVS, CNS and RS examinations were within normal limits.

**Figure 1:**
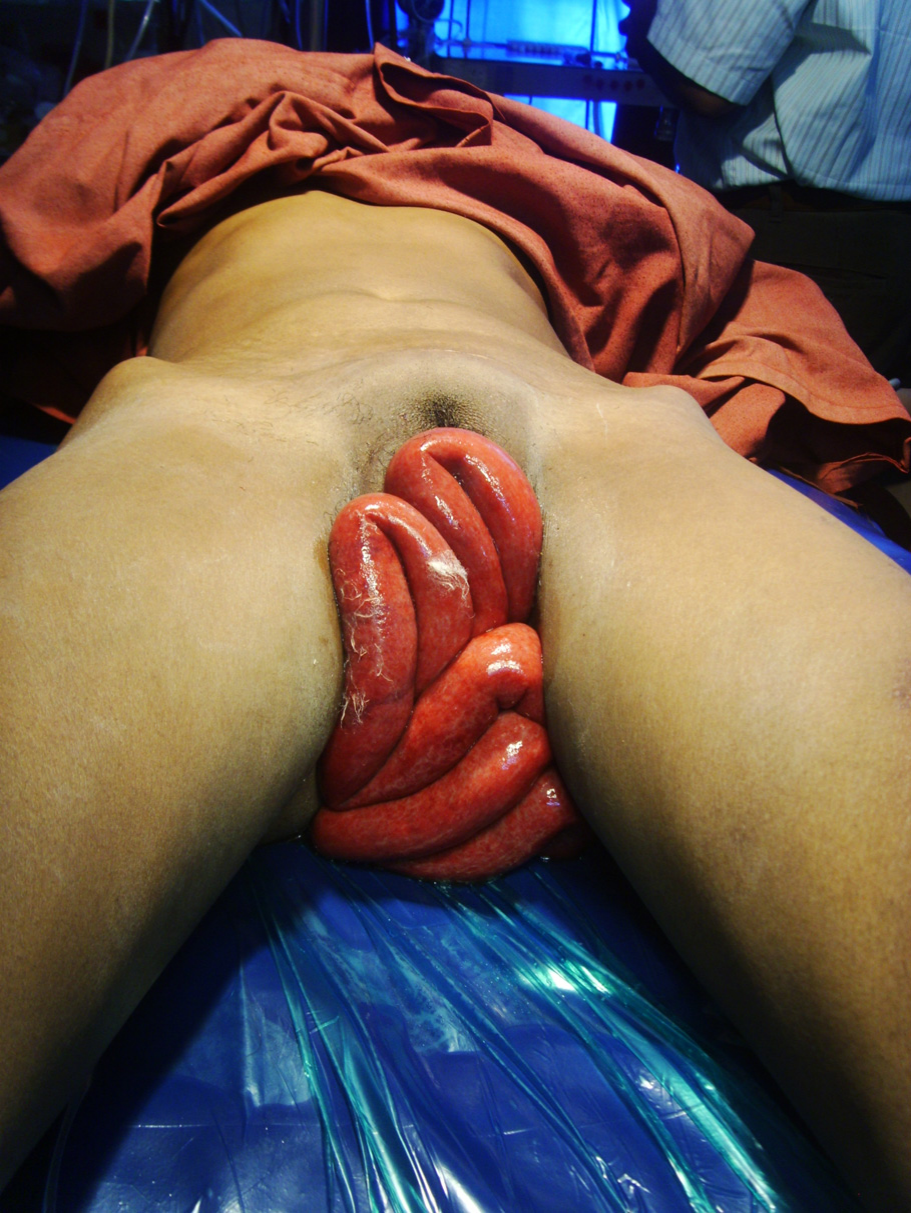
**Pelvic examination revealing around 1 meter of small bowel prolapse through the vagina**.

On **inspection**, around 1 meter of prolapsed small bowel was seen (Figure 1)

**Figure 2:**
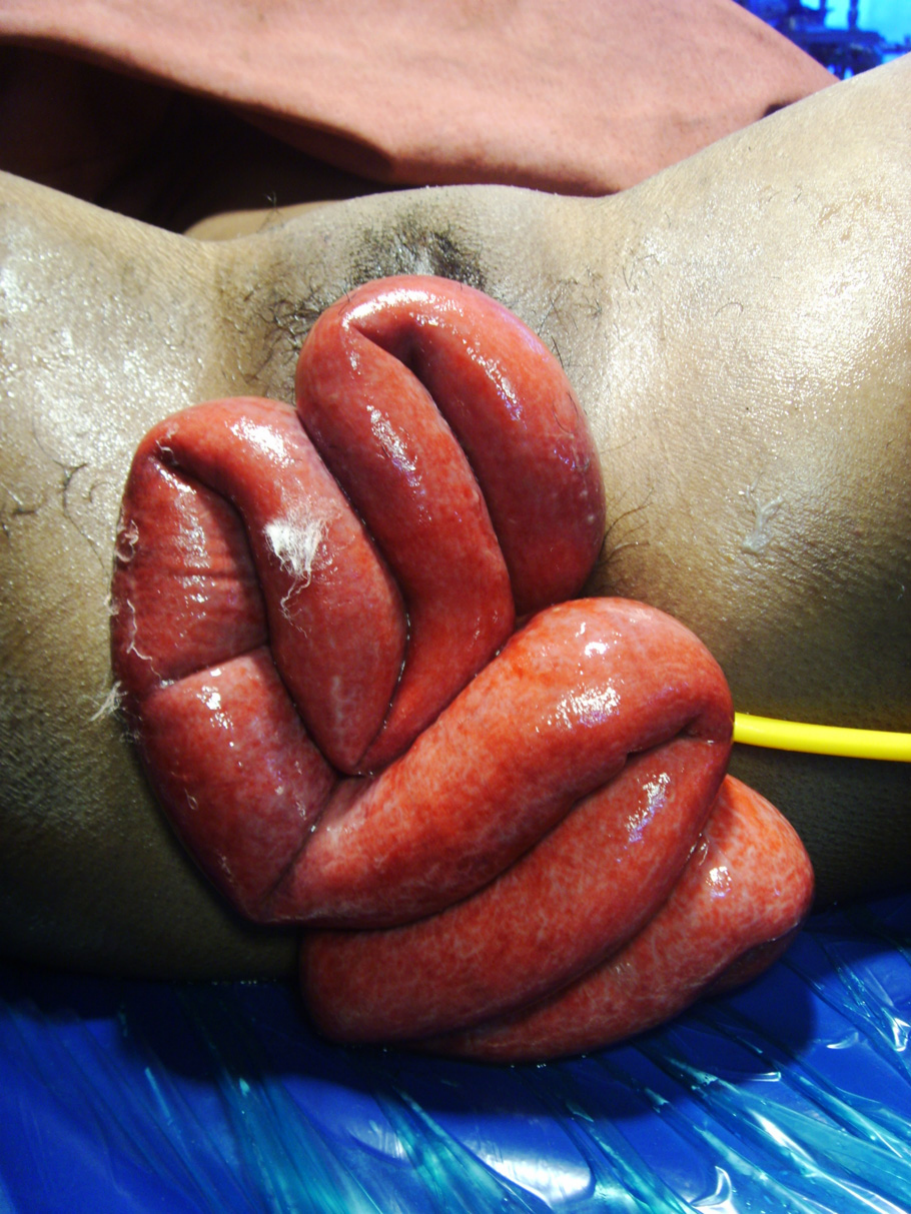
**On pelvic examination small bowel appeared non-ischemic but edematous, inflamed and thick walled**.

**Per-speculum examination **confirmed the prolapse of around 1 meter of the small bowel through a 5 cm fresh linear tear in vaginal vault. Vaginal vault was healthy with no signs of infection. On examination the small bowel appeared non-ischemic but edematous, inflamed and thick walled. (Figure 2)

### Investigations

Blood test showed an elevated WBC count of 16,300/mm^3^. Her blood group was B +ve. Urine tests were normal. Abdominal and chest X-ray were both unremarkable.

After resuscitation of the patient, she received broad spectrum antibiotics (ceftriaxone 1 gm + 500 mg metronidazole)

### Intra-operative findings

Laparotomy with lower midline vertical incision was performed which revealed a 5 cm fresh transverse tear in the vaginal vault without signs of infection. There was no ascites or growth or inflammatory disease in the pelvis. Rest of abdominal examination was normal.

Herniated small bowel is edematous, inflamed and non ischemic. All coils of intestine were reduced. 100% oxygen and wash with normal saline was given and the deficiency in the vaginal vault was closed using interrupted vicryl sutures.

She recovered uneventfully and was discharged on 7^th ^post operative day.

## Discussion

The incidence of vaginal rupture after any type of pelvic surgery is 0.03 percent with the reported incidence of cuff dehiscence after a hysterectomy being higher after laparoscopic hysterectomy compared with abdominal or vaginal hysterectomies [5,6]. Among the 7286 hysterectomies collection by Hur, an incidence of 0.14% was reported (total and subtotal), with a peak rate of 4.93% after laparoscopic hysterectomy [6] Another single institution case study (Loco on 3593 hysterectomies) reports a rate of 0.28%, without the evidence of statistical difference between different routes of access (trans-abdominal, trans-vaginal, or laparoscopic) or the presence of a closed or unclosed cuff [7].

Vaginal evisceration after trans-abdominal hysterectomy is rare in occurrence [2]. Review of literature over the years has been associated with vaginal rather than abdominal surgery [8]. Vaginal vault rupture with prolapse of small bowel during sexual intercourse after abdominal hysterectomy in pre-menopausal women is an extremely rare complication [3].

The risk groups for trans-vaginal bowel evisceration include the elderly, postmenopausal women and female patients after vaginal or laparoscopic hysterectomy [4]. Ramirez, on a review of the literature on 59 eviscerations, highlighted as risk factors: a postmenopausal state, trans-vaginal hysterectomy, and an increase in abdominal pressure [9].

Etiology of vaginal evisceration can be generally separated according to premenopausal or postmenopausal states. In postmenopausal women, evisceration can occur either spontaneously or more frequently in connection with an increase in intra abdominal pressure, induced by coughing, defecating or falling. In premenopausal patients, evisceration is usually preceded by vaginal trauma caused by rape, coitus, obstetric instrumentation or the insertion of the foreign bodies. Additional risk factors for vaginal evisceration include previous vaginal surgeries and enteroceles [4]. In young patients, sexual intercourse before the complete healing of the vaginal cuff is considered as the main trigger event, while in elderly patients, evisceration is a spontaneous event [7].

Establishing a diagnosis of coital vaginal trauma is often difficult as patients tend to give a misleading history. Coital trauma of vagina has been associated with multiparity. Nevertheless, the vaginal wall is designed to be extremely extended and the insertion of a penis alone should be unlikely to cause vaginal rupture [10].

There are several factors that may contribute to weakness at the vaginal apex. These are poor surgical technique, post operative wound or cuff infection, wound hematoma, resumption of sexual activity before complete healing, advanced age, previous radiotherapy, chronic steroid administration, trauma, previous vaginal surgery, a vulslava maneuver or straining during bowel movement [8]. Hysterectomy may enhance the risk of rupture as a complication of vaginal trauma, as the vagina is not supported by uterus [10].

Croak locates different sites of rupture between abdominal and vaginal hysterectomies, the former having lesions predominantly in the cuff, and the latter through a posterior wall enterocele, as might happen after radical pelvic operations for cancer [5]. Most of injuries reported occurred in posterior fornix because this is the most common direction of thrust during coitus and the upper vagina is unsupported except by bundles of connective tissue [10]. Evisceration can occur even after subtotal hysterectomy through the posterior fornix [1].

Bowel evisceration can lead to serious sequele, including peritonitis, bowel injury, necrosis and sepsis. The terminal ileum is most commonly protruding viscus, although other organs, such as omentum, salpinx, and epiploic appendices have also been described. Prompt surgical and medical intervention is required to prevent complications [8,10].

In our patient, there was no bleeding or free air in peritoneal cavity, supposedly because of the ruptured area of the vagina being immediately packed by the prolapsed intestine, which remained in the vagina from when the accident occurred. In the course of time, further prolapse might have developed when intra abdominal pressure grew high at evacuation.

The primary intervention for vaginal evisceration consists of stabilization, fluid therapy, wrapping the bowel with moist saline sponges, early antibiotic therapy, radiograph to exclude foreign bodies and prompt surgical intervention [8].

All the authors agree on the need for emergency reduction and repair. The operation can be accomplished either by a trans-abdominal (open or laparoscopic) technique, by a trans-vaginal route or by a combination of the two depending on the patient's condition and bowel viability at the time of treatment [8,11].

If the evisceration is associated with viable easily reducible bowel, lack of evidence of instrumentation historically and radiographically, the trans-vaginal approach consisting of a 2 layer closure of peritoneum and vagina should be considered [8]. To date, all the reported cases that have required bowel resection have been managed with exploratory laparotomy followed by repair of vaginal defect [12].

In our patient, there were signs of peritonitis, so we did exploratory laparotomy followed by reduction and repair.

The associated mortality rate of vaginal evisceration is 5.6%. However the incidence of morbidity is higher, when the bowel has become strangulated through vaginal defect [12].

## Conclusion

Vaginal evisceration is a potentially life threatening, rare and distressing complication of a very common gynecological procedure. So far reported cases of vaginal evisceration in premenopausal women tend to be rarer as compared to postmenopausal women; and are associated with sexual and obstetric trauma. It requires aggressive resuscitation and urgent surgical intervention to reduce morbidity and mortality.

Rarely do surgeons come across such unusual complications of hysterectomy, and therefore, the general surgeon must be aware of this extremely rare but potentially life threatening complication.

## Consent

Written informed consent was obtained from the patient for publication of this case report and any accompanying images. A copy of the written consent is available for review by the Editor-in-Chief of this journal.

## Abbreviations

DUB: Dysfunctional Uterine Bleeding; P2: Para 2; L2: Living 2

## Competing interests

The authors declare that they have no competing interests.

## Authors' contributions

RC, GC and NG performed the surgery. NG analyzed and interpreted the patients clinical data and was also a major contributor in writing the manuscript. MB, NB and SA assisted in overall study. All authors read and approved the final manuscript.
